# In vitro activity of antiamoebic drugs against clinical isolates of *Entamoeba histolytica *and *Entamoeba dispar*

**DOI:** 10.1186/1476-0711-3-27

**Published:** 2004-12-21

**Authors:** Devendra Bansal, Rakesh Sehgal, Yogesh Chawla, Ramesh Chander Mahajan, Nancy Malla

**Affiliations:** 1 Department of Parasitology, Post Graduate Institute of Medical Education & Research, Chandigarh, India; 2 Department of Hepatology, Post Graduate Institute of Medical Education & Research, Chandigarh, India

## Abstract

**Background:**

Amoebiasis is a major public health problem in tropical and subtropical countries. Although a number of antiamoebic agents are used for its treatment, yet the susceptibility data on clinical isolates of *Entamoeba histolytica *and *Entamoeba dispar *are not available. Therefore, the present study was aimed to assess the in vitro susceptibility of clinical isolates of *E. histolytica *and *E. dispar *to metronidazole, chloroquine, emetine and tinidazole.

**Methods:**

A total of 45 clinical isolates (15 *E. histolytica *and 30 *E. dispar*) were maintained in polyxenic cultures followed by monoxenic cultures. In vitro drug sensitivity (IC_50_) of clinical isolates and standard reference strain of *E. histolytica *(HM1: IMSS) was assessed by nitro blue tetrazolium (NBT) reduction assay after exposure to various concentrations of each drug.

**Results:**

The results showed that all clinical isolates had a higher IC_50 _compared to reference strain to all the four drugs. *E. histolytica *isolates appeared to be more susceptible [IC_50 _(μm) 13.2,26.3,31.2 and 12.4] compared to *E. dispar *isolates [IC_50_(μm) 15.6,28.9,32.8 and 13.2] and the reference strain of *E. histolytica *[IC_50 _(μm) 9.5, 15.5, 29.9 and 10.2] to the metronidazole, chloroquine, emetine and tinidazole respectively.

**Conclusions:**

The results indicate that till date, *Entamoeba *isolates in India do not seem to be resistant to the commonly used antiamoebic drugs.

## Background

*Entamoeba histolytica*, is the etiological agent of amoebic dysentery and amoebic liver abscess (ALA). Worldwide, 40–50 million symptomatic cases of amoebiasis occur annually and 70,000 to 100,000 deaths due to this infection [1]. There are two distinct, but morphologically identical species of *Entamoeba*: *Entamoeba histolytica*, which is pathogenic and *Entamoeba dispar*, which is non-pathogenic. *E. histolytica*, has the capacity to invade intestinal mucosa resulting in intestinal amoebiasis and cause extra intestinal amoebiasis [amoebic liver abscess (ALA)] [2].

Infection is primarily treated by instituting antiamoebic therapy. Drugs of choice for invasive amoebiasis are tissue active agents, like metronidazole, tinidazole and chloroquine or the more toxic emetine derivatives, including dehydroemetine. Metronidazole and tinidazole are derived from 5-nitroimdazole which kill the trophozoites by alterations in the protoplasmic organelles of the amoeba, but are ineffective in the treatment of cyst passers. Chloroquine is derived from 4-aminoquinolines, which acts on the vegetative forms of the parasite and kills it by inhibiting DNA synthesis. Emetine, a plant alkaloid, kills the trophozoites of *E. histolytica *mainly by inhibiting protein synthesis.

Indiscriminate use of drugs has led to an increase in the minimum inhibitory concentration (MIC) of these therapeutic agents [3]. Although, drug resistance to *E. histolytica *does not appear to be a serious problem, there are occasional reports of failure with metronidazole suggesting that this could probably be heralding the development of drug resistance clinically [4]. Recurrence of ALA even after treatment with metronidazole has been reported and parasites may survive in spite of adequate treatment [5]. However, differences in drug sensitivity between strains of *E. histolytica *have been reported, indicating that there may be a small percentage of amoebae which are either resistant to the drug or may even eventually become resistant due to abuse of antiamoebic agents [6]. Although, earlier studies have been focused on in vitro sensitivity of the only axenic strains of *E. histolytica *[7-9], yet to the best of our knowledge, studies on in vitro drug susceptibility studies on clinical isolates of *E. histolytica *and *E. dispar *have not been reported. Therefore, in the present study an attempt has been made to assess the in vitro activity of antiamoebic drugs (emetine, chloroquine, metronidazole and tinidazole) against clinical isolates of *E. histolytica *and *E. dispar*.

## Methods

### Clinical isolates

Forty-five isolates from patients attending the Out Patient Departments of Nehru hospital, attached to the Post Graduate Institute of Medical Education & Research, Chandigarh, India, identified earlier [10] as either *E. histolytica *(15) or *E. dispar *(30) by hexokinase isoenzyme analysis and by Techlab ELISA were used in the present study. These have been cultured in modified Boeck and Drbohlav (NIH) medium [11] followed by Robinson's medium [12].

### Standard reference strain (HM1: IMSS)

Reference strain of *E. histolytica *(HM1: IMSS) maintained axenically in TYI-S-33 medium was included as control [13].

### Preparation of antimicrobial agents

The drugs (metronidazole, chloroquine, emetine dihydrochloride and tinidazole) used in the study were procured as pure salt from Sigma-Aldrich Co., St. Louis, MO., 63178 USA. The stock solutions of drugs (each 0.1 M) were prepared in dimethyl sulphoxide (DMSO) [14] and stored at -20°C till use. The stock solutions were diluted in medium to the required concentration. A starting concentration used was 200 μM, which yielded a maximum concentration in the assay of 17.1 μg/ml metronidazole, 51.59 μg/ml chloroquine, 55.3 μg/ml emetine, and 24.7 μg/ml tinidazole.

### In vitro drug sensitivity assay

Drug sensitivity to all the compounds was carried out by nitroblue tetrazolium (NBT) reduction method [15]. Each clinical isolate was tested in duplicate along with the reference *E. histolytica *strain (HM1: IMSS). Amoebae were harvested from 24 hour old cultures and suspended in medium. The parasite count was adjusted to 3 × 10^5 ^parasites/ml in medium by haemocytometer [15].

The assay was carried out in microtiter plates (Grenier bio-one, Germany). Briefly, in row A 200 μl of drug and in all other rows (B-H) medium was added and doubling dilutions of the drug were performed down the plate. Final drug concentration in rows A-H was as follows: 100, 50, 25, 12.5, 6.25, 3.12, 1.6 and 0.8 (μM). Further 100 μl of parasite suspension (3 × 10^5^/well) was added to all the rows (A-H). Each test included control (without drug) and blank wells (medium only). The plates were incubated at 37°C for 4 hrs. The contents of the plates were discarded and washed with pre warmed Hank's balanced salt solution (HBSS pH 7.2). Thereafter, 100 μl of NBT/well in HBSS was added and the plates were incubated at 37°C for 45 min. followed by aspiration of the contents. Plates were then washed with HBSS twice and 200 μl/well of DMSO (100% v/v) was added. Following incubation at 37°C for 10 min, the optical density (OD) was measured in an ELISA recorder at 540 nm.

The percentage of non-viable organisms, which failed to metabolize NBT and therefore did not produce the dark blue formazan product, was determined by applying the following formula:

Percentage of non-viable organisms at each drug conc. =



### Statistical analysis

The mean IC_50 _values of all clinical isolates against the four drugs were compared with corresponding IC_50 _values of the reference *E. histolytica *strain (HM1: IMSS). Standard deviation (SD) was used to indicate the extent of variation around group mean values. The *p *value was calculated using the student's-t test.

## Results

**Figure 1:**
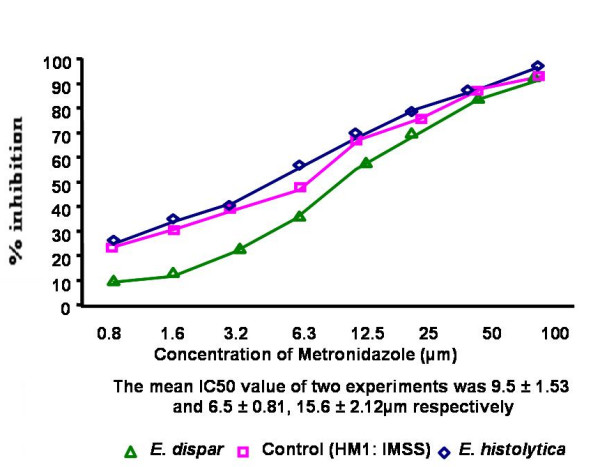
Percentage inhibition of *E. histolytica *(HM1: IMSS) and clinical isolates of *E. histolytica *and *E. dispar *by metronidazole

**Figure 2:**
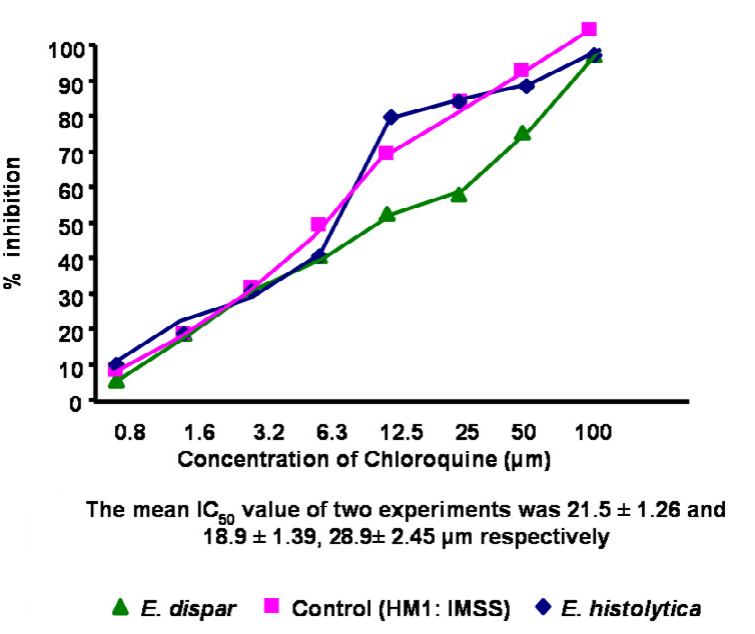
Percentage inhibition of *E. histolytica *(HM1: IMSS) and clinical isolates of *E. histolytica *and *E. dispar *by chloroquine

**Figure 3:**
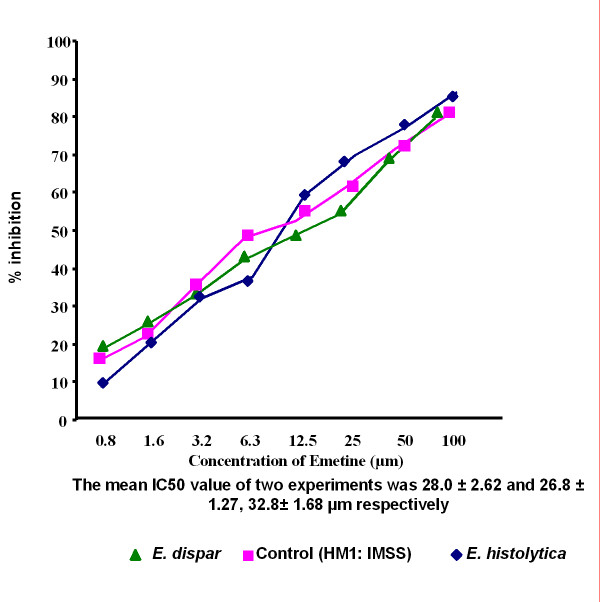
Percentage inhibition of *E. histolytica *(HM1: IMSS) and clinical isolates of *E. histolytica *and *E. dispar *by emetine

**Figure 4:**
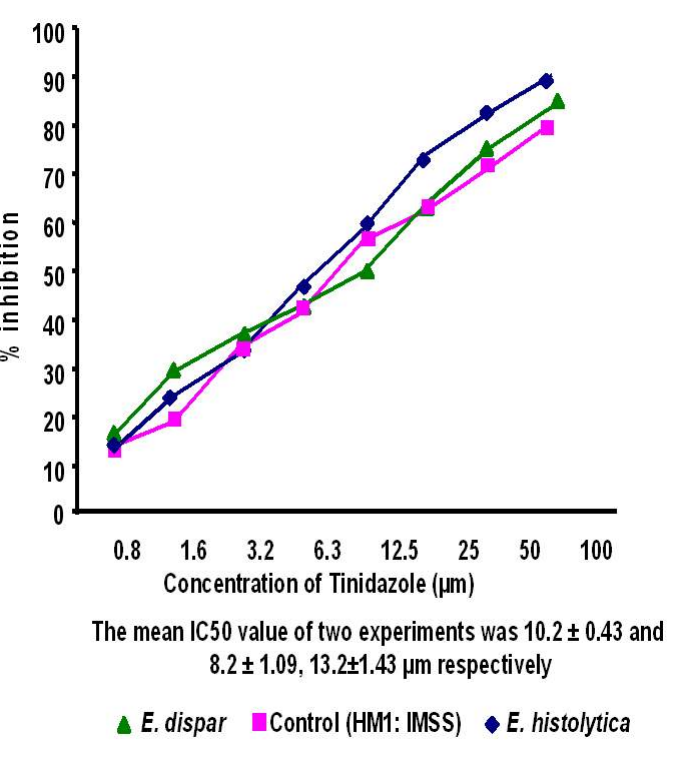
Percentage inhibition of *E. histolytica *(HM1: IMSS) and clinical isolates of *E. histolytica *and *E. dispar *by tinidazole

The IC_50 _values of emetine, chloroquine, metronidazole and tinidazole for the 45 clinical isolates [15 *E. histolytica *and 30 *E. dispar*] and the reference strain HM1: IMSS were determined by the NBT reduction assay. The mean IC_50 _values were significantly higher (*P *< 0.001) in *E. dispar *isolates to all the four antiamoebic drugs as compared to the *E. histolytica *isolates and the reference *E. histolytica *strain (Table 1 & Figures 1–4).

## Discussion

Treatment failure among amoebiasis patients often raises the possibility of drug resistance [16]. In the present study the 15 *E. histolytica *and 30 *E*. *dispar *clinical isolates maintained by in vitro cultivation in monoxenic medium were subjected to drug susceptibility tests against four antiamoebic drugs: metronidazole, chloroquine, emetine and tinidazole by NBT reduction assay. *E. histolytica *reference strain (HM1: IMSS) was also included in each set of experiments.

Results showed a significant difference in drug sensitivity in clinical isolates as compared to the reference strain with all the four drugs. The mean IC_50 _values (μm) of the *E. histolytica*/*E*. *dispar *isolates against metronidazole, chloroquine, emetine and tinidazole were 13.2/15.6, 26.3/28.9, 31.2/32.8 and 12.4/13.2 respectively. The IC_50 _values (μm) of the reference strain against all the four respective drugs were 9.5, 15.5, 29.9 and 10.2. Recently Upcroft & Upcroft [14] have reported that the MIC values of metronidazole ranges from 12.5–25 μm for laboratory-passaged *E. histolytica *strains. Adagu, et.al. [9] have shown the mean metronidazole IC_50 _value as 18.47 μm for the most susceptible isolates of *E. histolytica *with a > 30 μm value as the cut off for resistance. Burchard & Mirelman, studied in vitro sensitivity to metronidazole and emetine of non-pathogenic zymodemes and showed that all were similarly sensitive to both the drugs (1–10 μg/ml) [6]. In the present study, clinical isolates maintained in monoxenic culture were used to detect the in vitro sensitivity as earlier it has been concluded that bacterial flora associated with the amebae did not significantly interfere with the test performance and sensitivity values [6].

Although resistance to metronidazole has been reported against *Trichomonas vaginalis *[17], *Giardia *lamblia [18] and *Leishmania donovani *[19], yet to the best of our knowledge there is no documented resistance among clinical isolates of *E. histolytica *and *E*. dispar.

## Conclusion

The results of the present study are in agreement with previous findings [6,9,14], except that there was a significantly higher IC_50 _value of all four drugs to the clinical isolates as compared to the reference strain. *E. dispar *isolates showed higher IC_50 _values when compared to *E. histolytica *or reference strain. This is the first report of in vitro drug sensitivity pattern to clinical isolates of *E. histolytica *and *E. dispar*. There is definitely a need to monitor the random drug susceptibility among clinical isolates especially in context to widespread use of metronidazole and tinidazole, which are available over the counter in many countries. Increased awareness and continued surveillance for the possible emergence of resistance among clinical isolates is necessary for the ultimate prevention and control of amoebiasis.

## Authors' contributions

**DB**, carried the practical work mentioned in the manuscript.

**RS**, was responsible for formulation of the project and provided guidance time to time.

**YC**, he is clinician and carried clinical examination and proposed clinical diagnosis of the patient.

**RCM**, he guided the proposed work related to differentiation of *Entamoeba histolytica *and *Entamoeba dispar *and in vitro drug sensitivity.

**NM**, proposed the concept for this manuscript and guided the practical work and writing of the manuscript.

**Table 1:** Comparison between 1C_50 _value of clinical isolates (*E. histolytica *and *E. dispar*) vs reference strain (HM1: IMSS)

**COMPOUND**	**MEAN IC_50 _OF CLINICAL ISOLATES (μm ± SD)**		**IC_50 _VALUE OF REFERENCE STRAIN (μm ± SD)**
		
	***E. histolytica***	***E. dispar***	
**Metronidazole**	6.5 ± 0.81***^a^	15.6 ± 2.12***^c^	9.5 ± 1.53***^b^
**Chloroquine**	18.9 ± 1.39***^a^	28.9 ± 2.45***^c^	21.5 ± 1.26***^b^
**Emetine**	26.8 ± 1.27***^a^	32.8 ± 1.68***^c^	28.0 ± 2.62***^b^
**Tinidazole**	8.2 ± 1.09***^a^	13.2 ± 1.43***^c^	10.2 ± 0.43***^b^

**Results expressed as mean ± SD from two experiments conducted in duplicate**

**Student's t-test **[*** P < 0.001]

a = Eh Vs Ed **Eh – *E. histolytica***

b = Eh Vs C **Ed – *E. dispar***

c = Ed Vs C **C – Reference strain (HM1: IMSS)**
